# Analysis of hemi-uterus pregnancy outcomes in uterine malformations: a retrospective observational study

**DOI:** 10.1186/s12884-023-06136-w

**Published:** 2023-12-04

**Authors:** Liang Qian, Jiashan Ding, Lan Shi, Qing Yu, Jiawei Zhu, Anfeng Huang

**Affiliations:** 1 Department of Obstetrics, Hangzhou Women’s Hospital (Hangzhou Maternity and Child Health Care Hospital), Hangzhou, 310008 Zhejiang China; 2 Department of Obstetrics and Gynecology, the First Affiliated Hospital of Nanchang University, Nanchang, 330006 China; 3 Department of Fourth Clinical Medical College, Zhejiang Chinese Medical University, Hangzhou, China

**Keywords:** Congenital uterine anomalies, Cesarean section, Preterm birth, Pregnancy outcome, Premature rupture of membranes

## Abstract

**Background:**

The association between uterine malformations and adverse pregnancy outcomes is well recognized. However, studies on adverse pregnancy outcomes based on one kind of anatomical commonality between different uterine anomalies have not been reported. This study aimed to investigate pregnancy outcomes in pregnancies with uterine malformations when the pregnancy is confined to a hemi-uterus.

**Methods:**

A retrospective observational study of 336 women who gave birth at our hospital from 2015 to 2021 was performed. Women (*n* = 112) with a unicornuate, complete bicornuate, or didelphic uterus were set as the study group, and women (*n* = 224) with a normal uterus were set as the reference group. Maternal and neonatal outcomes were evaluated and compared between the two groups using Student’s t-test, one-way ANOVA, Chi-squared test, Yates correction for continuity, or Fisher’s exact test. Modified Poisson regression analyses were used to estimate the relationships between the hemi-uterus pregnancy and preterm birth, preterm premature rupture of membranes, and cesarean section rates by adjusting for potential confounders. A *P* value < 0.05 was considered significant.

**Results:**

Women in the study group had a higher history of spontaneous abortion (24.1% vs. 10.7%, *P* = 0.002) and intrauterine fetal death (5.4% vs. 0.4, *P* = 0.006). Compared with the reference group, the study group had significantly higher rates of assisted reproductive technology (9.4% vs. 2.2%, *P* = 0.001) and cord-around-the neck (54.5% vs. 29.9%, *P* = 0.000). Modified Poisson regression analyses showed that the study group was at higher risk for preterm birth (aRR, 6.8; 95% CI 2.7–16.7), preterm premature rupture of membranes (aRR, 14.1; 95% CI 3.2–62.5), malpresentation (aRR, 13.2; 95% CI 6.3–27.7), and cesarean section (aRR, 4.4; 95% CI 3.3–5.7).

**Conclusion:**

Women with a unicornuate, didelphic, or complete bicornuate uterus are at higher risk for some adverse pregnancy outcomes than those with a normal uterus.

## Background

The female uterus develops during the embryonic period from a pair of Müllerian ducts through differentiation, migration, fusion, and subsequent septal resorption [1]. Abnormalities in any of the developmental processes will result in malformations of the uterus. According to the ASRM Müllerian anomalies classification 2021 [2], uterus anomaly categories are identified by descriptive terminologies such as Müllerian agenesis, unicornuate uterus, uterus didelphys, bicornuate uterus, septate uterus, and complex anomalies. Some scholars [3–5] have classified these types into unification defects (unicornuate, bicornuate, and didelphic uterus) of the Müllerian ducts and canalization defects (septate uterus) due to resorption disorders of the midline septum for the facilitation of research.

The incidence of uterine anomalies was approximately 5.5% in an unselected population, 8% in infertile women, and 13.3% in those with miscarriages [4]. The true prevalence in the general population is unknown because some women are asymptomatic. The impact of uterine malformation is mainly reflected in the reproductive process. It has been widely recognized that canalization defects have the worst reproductive performance in early pregnancy, such as infertility and early miscarriage [6, 7]. In contrast, the main challenge of unification defects lies in maintaining pregnancy, as a series of obstetric and fetal complications often occur due to asymmetrical uterine morphology and diminished muscle mass [8].

**Fig. 1:**
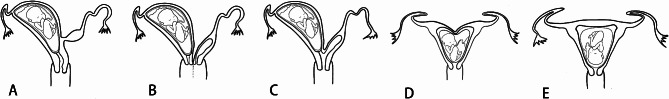
Different uterine morphologies during pregnancy. The fetus in **A**, **B**, and **C** are confined to one-half of the uterine cavity; in **F**, the fetus occupies the entire cavity, and in **D**, the extent of fetal occupation depends on the severity of the fundal indentation unicornuate uterus; **(B)** didelphic uterus; **(C)** complete bicornuate uterus; **(D)** incomplete bicornuate uterus; **(E)** normal uterus

This study aimed to assess the association of unification defects with pregnancy outcomes. Unlike previous publications, we considered the heterogeneity of the incomplete bicornuate uterus (Fig. 1) and excluded it from the study group. Therefore, this study is characterized by investigating the impact on reproductive, obstetric, and perinatal outcomes when pregnancy is confined to a hemi-uterus. Furthermore, we also performed a subgroup analysis to compare the pregnancy outcomes in each subgroup.

## Materials and methods

**Fig. 2:**
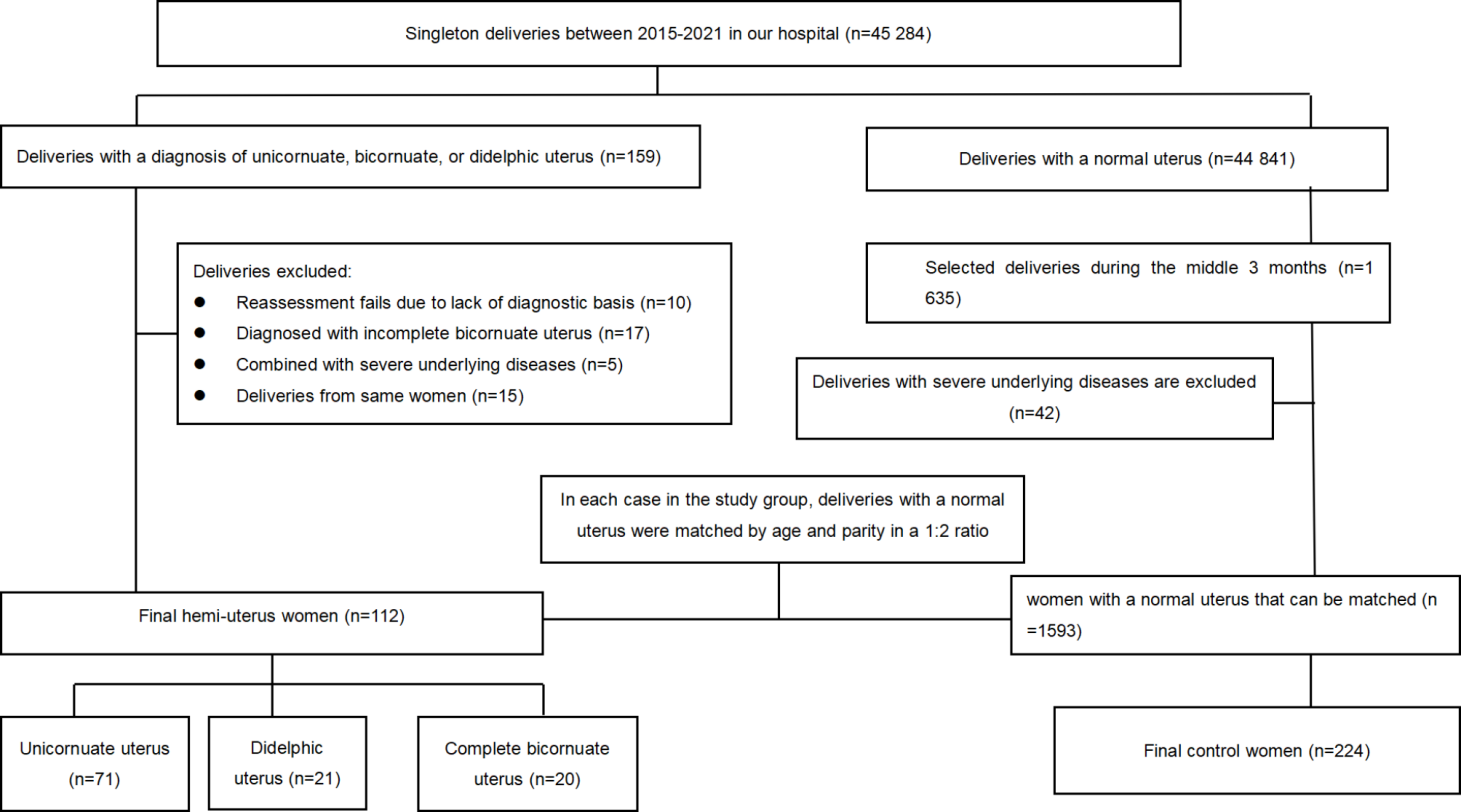
Study flowchart

We conducted a historical cohort study of women who gave birth at Hangzhou Women’s Hospital from January 1, 2015, until December 31, 2021 (Fig. 2). Only singleton pregnancies were included. The hospital’s electronic database identified all women diagnosed with unicornuate, complete bicornuate, or didelphic uterus. Women were diagnosed based on the classification system of the American Society of Reproductive Medicine [2]. Uterine abnormalities were diagnosed by physical examination, medical imaging (ultrasonography, hysterosalpingography), or surgery (hysteroscopy, laparoscopy, and laparotomy). The bicornuate uterus was defined as two partially separate uterine bodies, with external fundal indentation of > 1.0 cm, and some degree of unification at the inferior aspect. The complete bicornuate uterus was defined as separated uterine horns merged below the level of the internal cervical os. An incomplete bicornuate uterus was defined as horns merged above the internal cervical os [9].

We defined the study group as women with a unicornuate, complete bicornuate, or didelphic uterus and the reference group as women with a normal uterus. In the study group, for multiple deliveries by the same woman, only the first delivery in our hospital was selected for this study. Because the number of women with a normal uterus was vast, we randomly selected women in the middle three months from 2015 to 2021. For each case in the study group, women with a normal uterus were matched by age (± 2 years) and parity in a 1:2 ratio.

Women with severe underlying diseases, such as heart, respiratory, liver diseases, or coagulation abnormalities, were also excluded from our study. Maternal and neonatal data were mainly obtained by reviewing medical records and telephone interviews. The 2011 International Standard Classification of Education was used to categorize the education level into nine levels ranging from ISCED levels 0 to 9 [10].

The pregnancy and perinatal outcomes were defined as follows: spontaneous abortion (SA) refers to the loss of pregnancy naturally before 20 weeks of gestation; recurrent pregnancy loss refers to two or more consecutive spontaneous abortions; intrauterine fetal death (IUFD) is fetal death after 20 weeks of gestation but before the onset of labor; preterm birth (PTB) refers to childbirth at least 20 but before 37 gestational weeks; oligohydramnios refers to amniotic fluid index ≤ 5 cm or single deepest vertical pocket < 2 cm [11]; cord-around-the neck refers to the umbilical cord becomes wrapped around the fetal neck 360 degrees; small for gestational age (SGA) refers to birth weight below the 10th percentile for the gestational age using local gender-specific population-based curves; severe small for gestational age refers to birth weight below the 3rd percentile for the gestational age using local gender-specific population-based curves.

### Statistical analyses

Data analysis was performed with the SPSS version 25.0 package (Chicago, IL). Descriptive statistics are presented as the means and standard deviations (SDs) and as percentages for the enumerated data. Differences in the means between groups and subgroups were analyzed using Student’s t-test and one-way ANOVA. Counts and proportions were used for the categorical variables. The Chi-squared test, Yates correction for continuity, or Fisher’s exact test were performed to compare the categorical variables. Modified Poisson regression analyses were used to estimate the relationships between the hemi-uterus pregnancy and adverse pregnancy outcomes by adjusting for potential confounders. A *P* value < 0.05 was considered significant.

### Ethics Statement

This study was approved by the Ethics Committee of Hangzhou Women’s Hospital (date of approval: May 17, 2022; reference number: 2022 Medical Ethics Review K NO. (05)-01; Hangzhou, China). The Ethics Committee of Hangzhou Women’s Hospital granted a patient consent exemption because this retrospective study was harmless to the patients and contained no personal data.

## Results

A total of 336 women were included in our study. There were 112 women in the study group, including 71 women with a unicornuate uterus, 21 women with a didelphic uterus, and 20 women with a complete bicornuate uterus. A total of 224 women in a normal uterus served as the reference group. The baseline characteristics of the two groups are shown in Table 1. Maternal age, body mass index (BMI), and level of education were statistically similar between the two groups. Women in the study group had a higher rate of history of spontaneous abortion (24.1% vs. 10.7%, *P* = 0.002) and IUFD (5.4% vs. 0.4%, *P* = 0.006).

**Table 1:** Comparison of baseline characteristics between study and reference groups

	Study group (*n* = 112)	Reference group (*n* = 224)	*P* value
Maternal age (years)	29.3 ± 3.4	29.3 ± 3.2	0.972^&^
BMI (kg/m^2^)	25.7 ± 2.6	26.0 ± 3.2	0.425^&^
Parity			
1	91	182	
2	20	40	
3	1	2	
Level of education (%/*n*)			
ISCED level 1–3	3.6 (4)	1.3 (3)	0.228^*^
ISCED level 4–5	27.7 (31)	22.7 (51)	0.323^**#**^
ISCED level 6	57.1 (64)	59.8 (134)	0.638^**#**^
ISCED level 7–8	11.6 (13)	16.0 (36)	0.274^**#**^
SA history (%/*n*)	24.1 (27)	10.7 (24)	0.002^**#**^
RSA history (%/*n*)	1.8 (2)	0.4 (1)	0.259^*^
EP history (%/*n*)	5.4 (6)	2.2 (5)	0.233^§^
IUFD history (%/*n*)	5.4 (6)	0.4 (1)	0.006^*^

Data are presented as percent (numbers) or mean ± SD

BMI, body mass index; ISCED, international standard classification of education; SA, spontaneous abortion; RSA, recurrent spontaneous abortion; EP, ectopic pregnancy; IUFD, intrauterine fetal death

^#^ Chi-squared test ^*^Fisher’s exact test ^§^Yates correction for continuity

^&^Student’s t-test

Compared with the reference group, the study group had significantly higher rates of assisted reproductive technology (ART) (9.4% vs. 2.2%, *P* = 0.001), CAN (54.5% vs. 29.9%, *P* = 0.000), manual placenta removal (9.8% vs. 4.0%, *P* = 0.034), and CAN three times (7.1% vs. 0.4%, *P* = 0.001). The gestational age at birth was significantly earlier in the study group than in the reference group (37.9 ± 1.6 vs. 39.3 ± 1.2, *P* = 0.000), even when preterm births were removed (38.6 ± 0.9 vs. 39.4 ± 1.0, *P* = 0.000) (Table 2).

**Table 2:** Comparison of pregnancy characteristics between study and reference groups

	Study group (*n* = 112)	Reference group (*n* = 224)	*P* value
ART (%)	9.4 (12)	2.2 (5)	0.001^#^
Gestation age at birth (weeks)	37.9 ± 1.6	39.3 ± 1.2	0.000^&^
Gestation age at birth^a^ (weeks)	38.6 ± 0.9	39.4 ± 1.0	0.000^&^
Cervical incompetence	1/112	0/224	/
PROM (%/*n*)	21.4 (24)	25.4 (57)	0.417^#^
CAN (%/*n*)	54.5 (61)	29.9 (67)	0.000^#^
CAN three times (%/*n*)	7.1 (8)	0.4 (1)	0.001^§^
Manual placenta removal (%/*n*)	9.8 (11)	4.0 (9)	0.034^#^
Postpartum hemorrhage (%/*n*)	2.7 (3)	4.5 (10)	0.617^§^
Uterotonic usage (%/*n*)	40.2 (45)	37.5 (84)	0.634^#^
Oligohydramnios (%/*n*)	3.6 (4)	0.9 (2)	0.098^§^
Hypertensive disorders (%/*n*)	4.5 (5)	6.7 (15)	0.415^#^
Gestational diabetes (%/*n*)	17.0 (19)	14.7 (33)	0.594^#^
ICP (%/*n*)	2.7 (3)	1.3 (3)	0.404^*^
Placenta previa (%/*n*)	2.7 (3)	0.4(1)	0.110^*^
Placental abruption (%/*n*)	0.9 (1)	1.8 (4)	0.668^*^

Data are presented as percent (numbers) or mean ± SD

ART, assisted reproductive technology; PROM, premature rupture of membranes; CAN, cord-around-the neck; ICP, intrahepatic cholestasis of pregnancy

^a^ Excluding the preterm births

^#^ Chi-squared test ^*^Fisher’s exact test ^§^Yates correction for continuity

^&^Student’s t-test

Although there were no significant differences in the rates of SGA and severe SGA, the fetal birthweight of the study group was lower than that of the reference group (2880.0 ± 466.7 vs. 3234.5 ± 402.7, *P* = 0.000), including when prematurity was excluded (3015.0 ± 361.5 vs. 3247.0 ± 363.2, *P* = 0.000). There was no significant difference in the rate of NICU admission, but there were differences in the causes of NICU admission. The rate of neonatal sepsis was higher in the reference group (1.8% vs. 8.5%, *P* = 0.017), while the rate of NRDS was higher in the study group (5.4% vs. 0.9%, *P* = 0.031) (Table 3).

**Table 3:** Comparison of neonatal outcomes between study and reference group

	Study group (*n* = 112)	Reference group (*n* = 224)	*P* value
Birthweight (g)	2880.0 ± 466.7	3234.5 ± 402.7	0.000^&^
Birthweight^a^ (g)	3015.0 ± 361.5	3247.0 ± 363.2	0.000^&^
SGA (%/*n*)	16.1 (18)	12.1 (27)	0.308^#^
Severe SGA (%/*n*)	4.5 (5)	2.7 (6)	0.588^§^
Macrosomia (%/*n*)	0	1.8 (4)	
Apgar 5 min 7 (%/*n*)	0.9 (1)	0	
Neonatal sepsis (%/*n*)	1.8 (2)	8.5 (19)	0.017^#^
NRDS (%/*n*)	5.4 (6)	0.9 (2)	0.031^§^
Perinatal mortality	0	0	
Admission to NICU (%/*n*)	15.2 (17)	10.3 (23)	0.190^#^

Data are presented as percent (numbers) or mean ± SD

SGA, small for gestational age; NRDS, neonatal respiratory distress; NICU, neonatal intensive care unit;

^a^ Excluding the premature babies

^#^ Chi-squared test ^*^Fisher’s exact test ^§^Yates correction for continuity

^&^Student’s t-test

In a modified Poisson regression analysis model, after controlling for possible factors affecting interests, the study group was discovered to be at higher risk for PTB (aRR, 6.8; 95% CI 2.7–16.7), preterm premature rupture of membranes (PPROM) (aRR, 14.1; 95% CI 3.2–62.5), malpresentation (aRR, 13.2; 95% CI 6.3–27.7) and cesarean section (CS) (aRR, 4.4; 95% CI 3.3–5.7). The study group was still at higher risk for CS (aRR, 4.3; 95% CI 3.2–5.9) even without the multiparas (Table 4).

**Table 4:** Modified Poisson regression analysis for some important adverse outcomes in hemi-uterus pregnancy compared to the normal uterus

	Study group (*n* = 112)	Control group (*n* = 224)	*P* value		Unadjusted RR (95%CI)	Adjusted RR (95%CI)	*P* value
PTB (%/*n*)	18.8 (21)	2.8 (7)		0.000	7.0(2.9–16.9)	6.8^a^ (2.7–16.7)	0.000
PPROM (%/*n*)	11.6 (13)	0.9 (2)		0.001	13.0(3.0-56.6)	14.1^b^ (3.2–62.5)	0.001
Malpresentation (%/*n*)	45.5 (51)	3.1 (7)		0.000	14.6(6.8–31.1)	13.2^c^ (6.3–27.7)	0.000
CD (%/*n*)	90.2 (101)	21.0(47)		0.000	4.3(3.3–5.6)	4.4^d^ (3.3–5.7)	0.000
CD^e^ (%/*n*)	87.9 (80/91^e^)	17.0 (38/182^e^)		0.000	4.2 (3.1–5.6)	4.3^d^ (3.2–5.9)	0.000

Data are presented as percent (numbers) or mean ± SD

RR, risk ratio; CI, confidence interval; PTB, preterm birth, PPROM, preterm premature rupture of membranes; CS, cesarean section

^a^ Adjusted for age, BMI, level of education; ART, PROM, hypertensive disorders, gestational diabetes; placenta previa; placenta abruption;

^b^ Adjusted for age, BMI, level of education, hypertensive disorders, gestational diabetes;

^c^ Adjusted for ages, BMI, parity, birthweight, placenta previa;

^d^ Adjusted for age, ART, BMI, level of education, hypertensive disorders, gestational diabetes; ICP, placenta previa; placenta abrupt

^e^ Represent the number limited to nulliparous women

In the subgroup analysis, there were no statistically significant differences in baseline characteristics, obstetric characteristics, and neonatal outcomes, except in the rate of SGA (14.1% vs. 38.1% vs. 0%, *P* = 0.003) and malpresentation (54.9% vs.38.1% vs. 20.0%, *P* = 0.016), which were lower in the complete bicornuate group than in the other two subgroups (Table 5).

**Table 5:** Comparison of baseline characteristics, pregnancy, and neonatal outcomes between subgroups

	Unicornis (*n* = 71)	Didelphis (*n* = 21)	Complete bicornis (*n* = 20)	*P* value
Maternal age(years)	29.4 ± 3.4	28.5 ± 2.9	30.0 ± 3.4	0.361^&^
BMI (kg/m^2^)	25.6 ± 2.3	26.2 ± 3.5	25.5 ± 2.8	0.613^&^
Gravidity	1.8 ± 0.9	2.0 ± 1.0	1.7 ± 0.7	0.318^&^
Parity	1.2 ± 0.4	1.1 ± 0.4	1.7 ± 0.7	0.318^&^
ART (%/*n*)	11.3 (8)	4.8 (1)	15.0 (3)	0.554^*^
SA history (%/*n*)	24.0 (16)	28.6 (6)	25.0 (5)	0.850^#^
Gestation age at birth (weeks)	37.9 ± 1.7	37.8 ± 1.6	38.1 ± 1.5	0.851^&^
PTB (%/*n*)	15.5 (11)	28.6 (6)	20.0 (4)	0.366^*^
CS (%/*n*)	93.0 (66)	90.5 (19)	80.0 (16)	0.201^*^
CS^b^ (%/*n*)	91.2(52/57^a^)	88.9 (16/18^a^)	75 (12/16^a^)	0.219^*^
Malpresentation (%/*n*)	54.9 (39)	38.1 (8)	20.0 (4)	0.016^#^
PPROM (%/*n*)	8.5 (6)	23.8 (5)	10.0 (2)	0.218^*^
CAN (%/*n*)	53.5 (38)	57.1 (12)	55.0 (11)	0.957^#^
Birthweight (g)	2874.8 ± 443.6	2781.9 ± 523.0	3001.5 ± 483.0	0.321^&^
SGA (%/*n*)	14.1 (10)	38.1 (8)	0(0)	0.003^*^

Data are presented as percent (numbers) or mean ± SD

BMI, body mass index; ART, assisted reproductive technology; SA, spontaneous abortion; PTB, preterm birth; CS, cesarean section; PPROM, preterm premature rupture of membranes; CAN, cord-around-the neck; SGA, small for gestational age

^a^ Represent the number limited to nulliparous women

^#^ Chi-squared test ^*^Fisher’s exact test ^&^one-way ANOVA

Cesarean section was performed in 101 cases, with malpresentation (44.6%, 45/101) being the most common indication, followed by uterine anomalies (27.7%, 28/101) and previous cesarean Sect. (10.9%, 11/101). Cesarean section was performed in 79 cases when confined to primiparous women, with malpresentation (46.8%, 37/79) being the most common indication, followed by uterine anomalies (34.2%, 27/79). In comparing vaginal birth in primiparous women between the study group and the reference group, there was no statistically significant difference in the rates of induced labor, forceps delivery, postpartum hemorrhage, fetal birthweight, or the duration of labor (Table 6).

**Table 6:** Comparison of vaginal birth outcomes of primipara between the study and reference groups

	Study group (*n* = 11)	Reference group (*n* = 144)	*P* value
Gestation age at birth (weeks)	38.6 ± 1.9	39.4 ± 1.2	0.205^&^
Induction of labor (%/*n*)	27.3 (3)	56.9 (82)	0.111^§^
Birthweight (g)	3056.4 ± 493.6	3177.2 ± 377.5	0.319^&^
First stage labor duration (hours)	7.2 ± 4.2	9.7 ± 5.1	0.106^&^
Second stage labor duration (hours)	1.1 ± 0.8	1.4 ± 1.0	0.313^&^
Forceps delivery (%/*n*)	9.1(1)	9.0 (13)	1.000^*^
Postpartum hemorrhage (%/*n*)	9.1(1)	4.9 (7)	0.453^*^
Uterotonic usage (%/*n*)	45.5(5)	32.6 (47)	0.592^§^
Admission to NICU (%/*n*)	18.2 (2)	12.5(18)	0.940^§^

Data are presented as percent (numbers) or mean ± SD

NICU, neonatal intensive care unit

^*^Fisher’s exact test ^§^Yates correction for continuity ^&^Student’s t-test

## Discussion

Accompanied by the development of imaging techniques and ART availability, an increasing number of women with uterine anomalies are being detected [12]. It is important for clinicians to provide accurate information about this disease to these women. However, there are still some challenges in the research of this disease. Some studies compared all uterine malformations as a whole with the normal uterus group [13, 14], which creates a significant selection bias; some studies discussed individual malformations [9, 15] but were limited by small sample sizes, and some conducted meta-analyses by collecting published studies but were hampered by the heterogeneity of studies, such as inconsistency of classification systems and the discrepancy of study populations [16] Categorizing uterine malformations into canalization defects and unification defects can minimize the interference of studies caused by selection bias and insufficient sample size, but the differences in anatomical features between the incomplete bicornuate uterus and other malformations were easily neglected in previous studies [3–5]. In contrast to the complete bicornuate uterus, the severity of the fundal indentation of the incomplete bicornuate uterus is likely directly correlated with pregnancy outcome (Fig. 1). Therefore, this study excluded incomplete bicornuate uterus and retrospectively analyzed pregnancy outcomes when pregnancy was confined to a hemi-uterus. In addition, a subgroup analysis was performed to explore whether there were differences among the three subtypes, and the results showed that they did not differ significantly in baseline characteristics nor pregnancy outcomes, except for the incidence of SGA and malpresentation. The reason for the significantly lower incidence of SGA and malpresentation in the complete bicornis group compared with the other two subgroups is unclear. It may be due to the small sample size, which needs to be confirmed with a larger sample size in future studies. To some extent, the consistency of the conditions observed in these subtypes confirms the rationality of this classification.

Canalization defects are associated with the highest incidence of pregnancy loss and infertility [7] because of the presence of a septum that lacks blood supply and is unsuitable for embryo implantation [17]. This was clinically proven by improving the pregnancy rate and reducing the miscarriage rate through surgical treatment [18]. In our study, the higher rates of spontaneous abortion and IUFD history in the study group suggest that pregnancies in hemi-uterus may also be associated with a higher risk of fetal loss. The exact etiology remains unclear. A possible explanation is the hemi-uterus blood flow disturbance due to the absence or abnormality of uterine or ovarian arteries [19]. There was no significant difference in the rate of ectopic pregnancy history between the study and reference groups, which was consistent with previous studies [3].

Compared to canalization defects, the effects are more pronounced in late pregnancy in the case of hemi-uterus pregnancies. In terms of maintenance of pregnancy, the gestation age at birth was shorter in the study group, even when cases of PTB were excluded (Table 2). However, there was no difference in comparing the gestation age of the vaginal birth of primipara between the two groups (Table 6). The reason for this may be the high rate of cesarean section in the study group, with most pregnancies opting for cesarean section rather than waiting for the spontaneous onset of labor. In addition, our research suggested a notable risk of PTB, PPROM, and malpresentation in the study group. The causes of these phenomena were thought to be the limited capacity of the hemi-uterus, restricted distended uterine cavity, and weakened muscular support [20]. This theoretical explanation has been confirmed by experiments that established a causal relationship between uterine morphology and adverse outcomes. Li et al [20] found that the mean length of the hemi-uterus was 4.91 ± 0.55 cm, which was much smaller than the normal uterus, and demonstrated that women with longer uterine length (≥ 4.5 cm) were more likely to have term birth and that the uterine length and uterine cavity were independent protective factors for a better obstetric prognosis.

Several studies [21, 22] previously reported that some surgical techniques were used to expand the hemi-uterus and thus improved women’s pregnancy outcomes. However, the subjects for these procedures had severely malformed uteri and poor reproductive histories. This results in some selection bias. Moreover, it is worth noting that the rates of admission to the NICU and postpartum hemorrhage were not different. There was also no significant difference in the prevalence of pregnancy complications such as placenta previa, placental abruption, or gestational hypertension compared to the reference group. Considering that the hemi-uterus pregnancy did not cause serious obstetric or neonatal complications, routine surgical enlargement of the uterine cavity is not recommended in our opinion.

The CS rate in the study group was extremely high (90.2%, 101/112) compared to the reference group. Considering that the possibility of vaginal delivery may generally be higher in multiparas and previous CS may lead to CS again, the CS rates of two groups and subgroups were compared with the exclusion of multiparous women, and the results remained consistent (Tables 4 and 5). And the most common indication was also malpresentation. A significant number of women chose CS because of their malformed uteri. These women tend to be overly anxious and lacked confidence in having a normal birth [23]. Therefore, we have tried to compare the vaginal births of primiparous women in the study and reference groups (Table 6). There were no significant differences between the two groups in the duration of first-stage labor, second-stage labor, forceps delivery, postpartum hemorrhage, or uterotonic usage. This might indicate that the contractility and coordination of the hemi-uterus muscles are normative despite the lessened uterine musculature and abnormal uterine morphology. However, due to the small size of the vaginal births of primiparous women in the study group (11 cases), the conclusions reached are of limited persuasiveness. We recommend clinicians encourage women to try vaginal birth when adequately informed.

The innovative highlight of this study is an attempt to explore pregnancy outcomes in unicornuate, didelphic, and complete bicornuate uteri as a whole, characterized by their hemi-uterus pregnancy, which has not been considered in previous publications. The strengths of this study are its relatively large sample size, a small effect of confounding variables, and single-center records of maternal and infant parameters.

There are several deficiencies in our research. First, although these three subtypes have vital spatial commonalities at the anatomical level, it is not clear whether contralateral structures, such as the uterine anlage in women with unicornuate uterus [24], would impact the results. Second, for non-surgical women, the type of uterine anomaly was diagnosed primarily by ultrasound rather than the gold standard of magnetic resonance imaging. Despite reviewing each case and reassessing them to ensure the accuracy of the diagnosis, errors may exist. Third, since our study is a retrospective observational study and limited by the sample size, the poor number of some parameters led to restrictive refinement power, such as cervical incompetence, recurrent abortions, macrosomia, and some obstetric complications, convincing conclusions could not be drawn. Fourth, due to the absence of information about the etiology of women undergoing ART, it is not sure that the risk of infertility is increased in the study group.

## Conclusions

Unicornuate, didelphic, and complete bicornuate uteri share the same anatomical characteristic that the fetus is restricted to a hemi-uterus. This hemi-uterus pregnancy causes a significantly higher risk of PTB, PPROM, malpresentation, and CS. However, this hemi-uterus pregnancy does not seem to pose an increased risk of complications associated with vaginal birth.

## Data Availability

The datasets used and analyzed during the study are available from the corresponding author upon reasonable request.

## Abbreviations

IUFD: Intrauterine fetal death
CAN: Cord-around-the neck
SGA: Small for gestational age
PTB: Preterm birth
PPROM: Preterm premature rupture of membranes
